# (1*S*,2*R*,8*R*)-2,2-Dichloro-3,7,7,10-tetra­methyltricyclo­[6.4.0.0^1,3^]dodec-10-en-9-one

**DOI:** 10.1107/S1600536810040213

**Published:** 2010-10-23

**Authors:** Ahmed Benharref, Lahcen El Ammari, Moha Berraho

**Affiliations:** a Laboratoire de Chimie Biomoléculaires, Substances Naturelles et Réactivité, URAC16,Faculté des Sciences Semlalia, BP 2390 Bd My Abdellah, 40000 Marrakech, Morocco; b Laboratoire de Chimie du Solide Appliquée, Faculté des Sciences, Avenue Ibn Battouta BP 1014 Rabat, Morocco

## Abstract

The title compound, C_16_H_22_Cl_2_O, was synthesized from β-himachalene, which was isolated from essential oil of the Atlas cedar (*cedrus atlantica*). The asymmetric unit contains two independent mol­ecules, in each of which the six-membered ring shows a half-chair conformation, whereas the seven-membered ring displays a boat conformation. The dihedral angle between the two rings is slightly different in the two mol­ecules [63.22 (13) and 61.81 (14)°].

## Related literature

For the isolation of β-himachalene, see: Joseph & Dev (1968 ▶); Plattier & Teiseire (1974 ▶). For the reactivity of this sesquiterpene, see: Lassaba *et al.* (1998 ▶); Chekroun *et al.* (2000 ▶); El Jamili *et al.* (2002 ▶); Sbai *et al.* (2002 ▶); Dakir *et al.* (2004 ▶). For its biological activity, see: Daoubi *et al.* (2004 ▶).
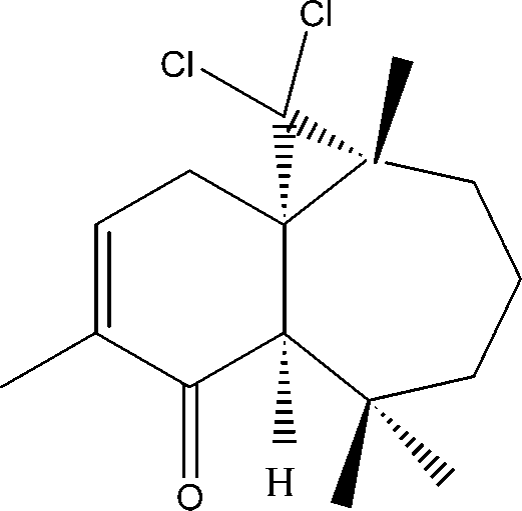


## Experimental

### 

#### Crystal data

C_16_H_22_Cl_2_O
*M* *_r_* = 301.24
Monoclinic, 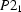

*a* = 6.6680 (7) Å
*b* = 18.7760 (16) Å
*c* = 12.7696 (12) Å
β = 90.430 (3)°
*V* = 1598.7 (3) Å^3^
*Z* = 4
Mo *K*α radiation
μ = 0.40 mm^−1^
*T* = 298 K
0.27 × 0.18 × 0.12 mm

#### Data collection

Bruker X8 APEXII CCD area-detector diffractometer
11524 measured reflections
5817 independent reflections
4348 reflections with *I* > 2σ(*I*)
*R*_int_ = 0.031

#### Refinement

*R*[*F* ^2^ > 2σ(*F* ^2^)] = 0.039
*wR*(*F* ^2^) = 0.094
*S* = 1.00
5817 reflections
351 parameters
1 restraint
H-atom parameters constrained
Δρ_max_ = 0.17 e Å^−3^
Δρ_min_ = −0.17 e Å^−3^
Absolute structure: Flack & Bernardinelli (2000 ▶), 1791 Friedel pairs
Flack parameter: 0.01 (5)

### 

Data collection: *APEX2* (Bruker, 2009 ▶); cell refinement: *SAINT* (Bruker, 2009 ▶); data reduction: *SAINT*; program(s) used to solve structure: *SHELXS97* (Sheldrick, 2008 ▶); program(s) used to refine structure: *SHELXL97* (Sheldrick, 2008 ▶); molecular graphics: *ORTEP-3 for Windows* (Farrugia, 1997 ▶); software used to prepare material for publication: *WinGX* (Farrugia, 1999 ▶).

## Supplementary Material

Crystal structure: contains datablocks I, global. DOI: 10.1107/S1600536810040213/im2235sup1.cif

Structure factors: contains datablocks I. DOI: 10.1107/S1600536810040213/im2235Isup2.hkl

Additional supplementary materials:  crystallographic information; 3D view; checkCIF report

## Figures and Tables

**Fig. 1.**
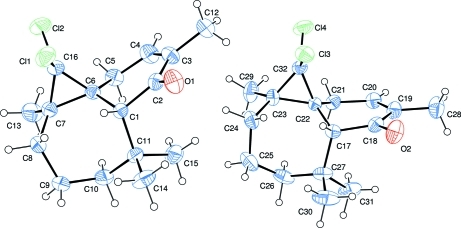
Molecular structure of the title compound with the atom-labelling scheme. Displacement ellipsoids are drawn at the 30% probability level. H atoms are represented as small spheres of arbitrary radii.

## supplementary crystallographic information

### Comment

The essential oil of the Atlas cedar (*cedrus atlantica*) consists mainly (50%) of a bicyclic hydrocarbon sesquiterpene called β-himachalene (Joseph & Dev 1968; Plattier & Teiseire 1974). The reactivity of this sesquiterpene has been studied extensively by our team in order to prepare new products having biological proprieties (Lassaba *et al.*, 1998; Chekroun *et al.*, 2000; El Jamili *et al.*, 2002; Sbai *et al.*, 2002; Dakir *et al.*, 2004). Indeed, these compounds were tested, using the food poisoning technique, for their potential antifungal activity against phytopathogen Botrytis cinerea (Daoubi *et al.*, 2004). Thus the action of one equivalent of dichlorocarbene, generated *in situ* from chloroform and in the presence of sodium hydroxide as base and n-benzyltriethylammonium chloride as catalyst, followed by epoxydation with *m*-chloroperbenzoic acid (*m*-CPBA) gives a mixture of two diasteroisomers:(1*S*,3*R*,8*S*,9*S*,10*R*)-2,2-dichloro-9,10-epoxy-3,7,7,10-tetramethyltricyclo[6.4.0.0^1,3^]dodecane (*X*, 70%) and (1*S*,3*R*,8*S*,9*R*,10*S*)-2,2-dichloro-9,10-epoxy-3,7,7,10-tetramethyltricyclo[6.4.0.0^1,3^]dodecane (*Y*, 30%) (Sbai *et al.*, 2002). By treatment of the majority isomer (*X*) with hydrochloric acid (see experimental) we obtained the title compound in 70% yield. The structure of (I) was established by ^1^H and ^13^C NMR and confirmed by its single crystal X-ray structure. The asymmetric unit contains two crystallographically independent molecules (Fig.1). Each molecule is built up from two fused six and seven-membered rings with the dichlorocyclopropyl ring at positions 1 and 2 in α-configuration. The six-membered ring shows a half chair conformation, whereas the seven-membered ring displays a boat conformation. In the first molecule (C1 to C16), the dihedral angle between the rings is 63.22 (13)°. The corresponding value in the second molecule (C17 to C31) is 61.81 (14)°. Owing to the presence of the Cl atoms, the absolute configuration could be fully confirmed to be (1*S*,2*R*,8*R*) for both molecules (Flack & Bernardinelli, 2000).

### Experimental

A solution is prepared by bubbling gaseous hydrochloric acid into 40 ml of chloroform for 1 minute. Then 1 g (3.30 mmol) of (1*S*,3*R*,8*S*,9*S*,10*R*)-2,2-dichloro-9,10-epoxy-3,7,7,10-tetramethyltricyclo[6.4.0.0^1,3^]dodecane (*X*) dissolved in 20 ml of chloroform, is added to this solution and the resulting mixture is stirred for 1 h. After evaporation of the solvent and chromatography of the residue obtained on a column of silica gel, using as eluent hexane-ethyl acetate (95/ 5), the corresponding enol is isolated (yield: 0.6 g, 1.98 mmol, 60%). A solution of the enol (0.5 g, 1.65 mmol) in dichloromethane (20 ml) was added to a suspention of the complex (CrO_3_: pyridine) (10 mmol) in another 45 ml of dichloromethane. The reaction mixture was stirred at room temperature for 1 h, then filtered through an alumina column. After evaporation of the solvent, the title compound, (1*S*,2*R*,8*R*)-2,2-Dichloro-3,7,7,10-tetramethyl-tricyclo[6.4.0.0^1,3^]dodec-10-en-9-one, is obtained (0.35 g, 70%). Crystals suitable for X-ray analysis were produced by recrystallization from *n*-pentane.

### Refinement

All H atoms were fixed geometrically and treated as riding with C—H = 0.96 Å (methyl),0.97 Å (methylene), 0.98Å (methine) with *U*_iso_(H) = 1.2U_eq _(methylene, methine) or *U*_iso_(H) = 1.5U_eq_ (methyl).

### Crystal data

**Table:**

C_16_H_22_Cl_2_O	*F*(000) = 640
*M**_r_* = 301.24	*D*_x_ = 1.252 Mg m^−^^3^
Monoclinic, *P*2_1_	Mo *K*α radiation, λ = 0.71073 Å
Hall symbol: P 2yb	Cell parameters from 11524 reflections
*a* = 6.6680 (7) Å	θ = 2.3–28.2°
*b* = 18.7760 (16) Å	µ = 0.40 mm^−^^1^
*c* = 12.7696 (12) Å	*T* = 298 K
β = 90.430 (3)°	Prism, colourless
*V* = 1598.7 (3) Å^3^	0.27 × 0.18 × 0.12 mm
*Z* = 4	

### Data collection

**Table:**

Bruker X8 APEXII CCD area-detector diffractometer	4348 reflections with *I* > 2σ(*I*)
Radiation source: fine-focus sealed tube	*R*_int_ = 0.031
graphite	θ_max_ = 28.2°, θ_min_ = 2.7°
φ and ω scans	*h* = −8→8
11524 measured reflections	*k* = −16→24
5817 independent reflections	*l* = −14→17

### Refinement

**Table:**

Refinement on *F*^2^	Secondary atom site location: difference Fourier map
Least-squares matrix: full	Hydrogen site location: inferred from neighbouring sites
*R*[*F*^2^ > 2σ(*F*^2^)] = 0.039	H-atom parameters constrained
*wR*(*F*^2^) = 0.094	*w* = 1/[σ^2^(*F*_o_^2^) + (0.0451*P*)^2^] where *P* = (*F*_o_^2^ + 2*F*_c_^2^)/3
*S* = 1.00	(Δ/σ)_max_ < 0.001
5817 reflections	Δρ_max_ = 0.17 e Å^−^^3^
351 parameters	Δρ_min_ = −0.17 e Å^−^^3^
1 restraint	Absolute structure: Flack & Bernardinelli (2000), 1791 Friedel pairs
Primary atom site location: structure-invariant direct methods	Flack parameter: 0.01 (5)

### Special details

**Table:**

Geometry. All e.s.d.'s (except the e.s.d. in the dihedral angle between two l.s. planes) are estimated using the full covariance matrix. The cell e.s.d.'s are taken into account individually in the estimation of e.s.d.'s in distances, angles and torsion angles; correlations between e.s.d.'s in cell parameters are only used when they are defined by crystal symmetry. An approximate (isotropic) treatment of cell e.s.d.'s is used for estimating e.s.d.'s involving l.s. planes.

### Fractional atomic coordinates and isotropic or equivalent isotropic displacement parameters (Å^2^)

**Table:**

	*x*	*y*	*z*	*U*_iso_*/*U*_eq_	
C4	0.2648 (4)	0.51700 (18)	0.1518 (2)	0.0665 (8)	
H4	0.3713	0.5344	0.1916	0.080*	
C5	0.3032 (4)	0.50170 (17)	0.0397 (2)	0.0566 (7)	
H5A	0.3645	0.4551	0.0335	0.068*	
H5B	0.3967	0.5366	0.0125	0.068*	
C6	0.1141 (4)	0.50357 (13)	−0.02434 (19)	0.0428 (5)	
C7	0.1190 (4)	0.50653 (15)	−0.1455 (2)	0.0555 (7)	
C13	0.3194 (5)	0.5040 (2)	−0.2006 (3)	0.0842 (11)	
H13A	0.3063	0.5251	−0.2688	0.126*	
H13B	0.3615	0.4553	−0.2077	0.126*	
H13C	0.4173	0.5298	−0.1603	0.126*	
C29	0.7542 (5)	0.3406 (2)	0.3751 (3)	0.0802 (10)	
H29A	0.7532	0.3825	0.3320	0.120*	
H29B	0.7934	0.3003	0.3338	0.120*	
H29C	0.8478	0.3469	0.4318	0.120*	
C30	0.1340 (6)	0.1455 (2)	0.4271 (3)	0.1001 (13)	
H30A	0.0623	0.1353	0.4903	0.150*	
H30B	0.1376	0.1036	0.3841	0.150*	
H30C	0.0677	0.1832	0.3898	0.150*	
C32	0.4841 (4)	0.36421 (13)	0.51889 (19)	0.0483 (6)	
Cl2	0.23340 (15)	0.64403 (4)	−0.07935 (7)	0.0867 (3)	
Cl3	0.24108 (11)	0.40022 (3)	0.53257 (6)	0.0636 (2)	
Cl4	0.65593 (14)	0.42095 (4)	0.58207 (7)	0.0779 (2)	
C1	−0.0602 (3)	0.46308 (12)	0.02462 (19)	0.0401 (5)	
H1	−0.1815	0.4787	−0.0126	0.048*	
C2	−0.0857 (4)	0.48562 (13)	0.1377 (2)	0.0478 (6)	
C3	0.0909 (4)	0.50787 (16)	0.1996 (2)	0.0577 (7)	
C8	−0.0460 (5)	0.46708 (15)	−0.2027 (2)	0.0635 (8)	
H8A	−0.0446	0.4806	−0.2760	0.076*	
H8B	−0.1744	0.4812	−0.1742	0.076*	
C9	−0.0246 (6)	0.38591 (16)	−0.1941 (2)	0.0753 (9)	
H9A	−0.1549	0.3645	−0.2070	0.090*	
H9B	0.0644	0.3698	−0.2490	0.090*	
C10	0.0552 (5)	0.35865 (15)	−0.0899 (2)	0.0681 (8)	
H10A	0.1938	0.3738	−0.0837	0.082*	
H10B	0.0560	0.3070	−0.0933	0.082*	
C11	−0.0502 (4)	0.37973 (13)	0.0114 (2)	0.0476 (6)	
C12	0.0569 (6)	0.52838 (19)	0.3121 (2)	0.0791 (10)	
H12A	0.1817	0.5263	0.3499	0.119*	
H12B	−0.0371	0.4960	0.3430	0.119*	
H12C	0.0043	0.5759	0.3151	0.119*	
C14	−0.2671 (5)	0.35177 (17)	0.0103 (3)	0.0793 (10)	
H14A	−0.2660	0.3008	0.0045	0.119*	
H14B	−0.3383	0.3718	−0.0483	0.119*	
H14C	−0.3322	0.3653	0.0741	0.119*	
C15	0.0620 (5)	0.34352 (16)	0.1020 (3)	0.0709 (9)	
H15A	0.0584	0.2928	0.0925	0.106*	
H15B	−0.0008	0.3557	0.1670	0.106*	
H15C	0.1989	0.3594	0.1032	0.106*	
C16	0.0640 (4)	0.57190 (14)	−0.0831 (2)	0.0545 (7)	
C17	0.3373 (3)	0.23635 (12)	0.52663 (18)	0.0416 (5)	
H17	0.2229	0.2643	0.5009	0.050*	
C18	0.2929 (4)	0.21847 (13)	0.6403 (2)	0.0491 (6)	
C19	0.4595 (4)	0.21347 (15)	0.7164 (2)	0.0566 (7)	
C20	0.6448 (4)	0.23164 (15)	0.6869 (2)	0.0541 (7)	
H20	0.7473	0.2279	0.7364	0.065*	
C21	0.6985 (4)	0.25743 (14)	0.5803 (2)	0.0504 (6)	
H21A	0.7596	0.2188	0.5415	0.060*	
H21B	0.7965	0.2954	0.5867	0.060*	
C22	0.5185 (4)	0.28455 (12)	0.51998 (19)	0.0394 (5)	
C23	0.5449 (4)	0.32811 (15)	0.41890 (19)	0.0503 (6)	
C24	0.3892 (5)	0.31700 (17)	0.3332 (2)	0.0620 (8)	
H24A	0.4055	0.3534	0.2801	0.074*	
H24B	0.2564	0.3223	0.3627	0.074*	
C25	0.4058 (6)	0.2439 (2)	0.2822 (2)	0.0831 (10)	
H25A	0.5018	0.2470	0.2256	0.100*	
H25B	0.2768	0.2320	0.2513	0.100*	
C26	0.4694 (5)	0.18318 (19)	0.3547 (3)	0.0788 (10)	
H26A	0.6073	0.1919	0.3757	0.095*	
H26B	0.4690	0.1398	0.3135	0.095*	
C27	0.3494 (4)	0.16840 (15)	0.4547 (2)	0.0612 (8)	
C28	0.4103 (5)	0.1891 (2)	0.8254 (3)	0.0821 (10)	
H28A	0.5294	0.1903	0.8681	0.123*	
H28B	0.3590	0.1414	0.8230	0.123*	
H28C	0.3112	0.2201	0.8549	0.123*	
C31	0.4526 (6)	0.10533 (17)	0.5119 (3)	0.0908 (11)	
H31A	0.5875	0.1184	0.5304	0.136*	
H31B	0.4548	0.0646	0.4665	0.136*	
H31C	0.3795	0.0940	0.5742	0.136*	
O1	−0.2520 (3)	0.48319 (13)	0.17818 (16)	0.0732 (6)	
O2	0.1224 (3)	0.20740 (13)	0.66801 (17)	0.0771 (6)	
Cl1	−0.18216 (13)	0.60662 (4)	−0.08502 (7)	0.0734 (2)	

### Atomic displacement parameters (Å^2^)

**Table:**

	*U*^11^	*U*^22^	*U*^33^	*U*^12^	*U*^13^	*U*^23^
C4	0.0487 (17)	0.085 (2)	0.065 (2)	−0.0084 (15)	−0.0153 (14)	−0.0067 (17)
C5	0.0387 (14)	0.0655 (18)	0.0656 (18)	−0.0037 (12)	−0.0004 (12)	−0.0059 (14)
C6	0.0402 (13)	0.0434 (13)	0.0447 (14)	−0.0043 (10)	−0.0006 (11)	−0.0034 (11)
C7	0.0677 (18)	0.0501 (15)	0.0489 (16)	−0.0119 (13)	0.0092 (13)	−0.0005 (12)
C13	0.092 (3)	0.089 (3)	0.072 (2)	−0.019 (2)	0.0332 (19)	−0.0136 (19)
C29	0.072 (2)	0.100 (3)	0.070 (2)	−0.0123 (19)	0.0127 (17)	0.0220 (19)
C30	0.077 (2)	0.087 (3)	0.137 (3)	−0.019 (2)	−0.014 (2)	−0.052 (3)
C32	0.0542 (15)	0.0398 (13)	0.0506 (16)	0.0004 (11)	−0.0125 (12)	0.0009 (11)
Cl2	0.1082 (7)	0.0573 (5)	0.0947 (6)	−0.0378 (5)	0.0078 (5)	−0.0006 (4)
Cl3	0.0704 (5)	0.0544 (4)	0.0658 (4)	0.0229 (3)	−0.0103 (3)	−0.0051 (3)
Cl4	0.0927 (6)	0.0486 (4)	0.0918 (6)	−0.0169 (4)	−0.0318 (4)	−0.0002 (4)
C1	0.0353 (13)	0.0399 (13)	0.0449 (14)	0.0020 (10)	−0.0076 (10)	0.0039 (11)
C2	0.0454 (15)	0.0471 (14)	0.0511 (16)	0.0094 (11)	0.0035 (12)	0.0059 (12)
C3	0.0539 (18)	0.0667 (18)	0.0522 (16)	0.0042 (14)	−0.0065 (13)	−0.0101 (14)
C8	0.089 (2)	0.0588 (17)	0.0430 (15)	−0.0148 (15)	−0.0073 (14)	−0.0008 (13)
C9	0.118 (3)	0.0553 (18)	0.0527 (17)	−0.0167 (18)	−0.0016 (17)	−0.0117 (14)
C10	0.091 (2)	0.0446 (16)	0.069 (2)	0.0051 (15)	−0.0035 (16)	−0.0075 (14)
C11	0.0488 (15)	0.0406 (13)	0.0532 (15)	−0.0014 (11)	−0.0020 (12)	0.0025 (11)
C12	0.105 (3)	0.080 (2)	0.0529 (18)	−0.003 (2)	−0.0018 (17)	−0.0074 (16)
C14	0.075 (2)	0.0495 (17)	0.114 (3)	−0.0145 (15)	−0.0048 (19)	0.0074 (17)
C15	0.081 (2)	0.0552 (17)	0.076 (2)	0.0137 (15)	−0.0104 (17)	0.0120 (15)
C16	0.0626 (18)	0.0459 (14)	0.0549 (17)	−0.0149 (13)	−0.0024 (13)	−0.0026 (13)
C17	0.0348 (13)	0.0408 (13)	0.0490 (15)	0.0067 (10)	−0.0050 (10)	−0.0034 (11)
C18	0.0430 (15)	0.0421 (14)	0.0624 (17)	0.0073 (11)	0.0050 (12)	0.0054 (12)
C19	0.0551 (17)	0.0587 (17)	0.0559 (17)	0.0108 (13)	−0.0002 (13)	0.0135 (13)
C20	0.0510 (16)	0.0544 (15)	0.0566 (17)	0.0087 (12)	−0.0141 (13)	0.0066 (13)
C21	0.0460 (15)	0.0466 (14)	0.0584 (17)	0.0029 (11)	−0.0054 (12)	0.0061 (12)
C22	0.0378 (13)	0.0395 (12)	0.0410 (13)	0.0040 (10)	−0.0019 (10)	−0.0007 (10)
C23	0.0494 (16)	0.0582 (16)	0.0433 (14)	0.0008 (12)	−0.0001 (12)	0.0077 (13)
C24	0.070 (2)	0.0738 (19)	0.0416 (15)	0.0087 (15)	−0.0054 (13)	0.0035 (15)
C25	0.102 (3)	0.098 (3)	0.0487 (18)	0.011 (2)	−0.0075 (17)	−0.0172 (18)
C26	0.088 (2)	0.073 (2)	0.076 (2)	0.0114 (18)	0.0056 (18)	−0.0394 (18)
C27	0.0563 (17)	0.0455 (15)	0.082 (2)	−0.0012 (12)	−0.0020 (15)	−0.0182 (15)
C28	0.090 (2)	0.090 (3)	0.066 (2)	0.015 (2)	0.0108 (18)	0.0236 (19)
C31	0.097 (3)	0.0443 (17)	0.131 (3)	0.0114 (18)	0.003 (2)	−0.010 (2)
O1	0.0502 (12)	0.1049 (17)	0.0647 (13)	0.0081 (11)	0.0127 (10)	0.0001 (12)
O2	0.0439 (12)	0.0959 (17)	0.0917 (16)	0.0044 (11)	0.0137 (10)	0.0280 (13)
Cl1	0.0823 (6)	0.0530 (4)	0.0846 (5)	0.0118 (4)	−0.0146 (4)	0.0132 (4)

### Geometric parameters (Å, °)

**Table:**

C4—C3	1.326 (4)	C11—C15	1.532 (4)
C4—C5	1.484 (4)	C11—C14	1.538 (4)
C4—H4	0.9300	C12—H12A	0.9600
C5—C6	1.498 (4)	C12—H12B	0.9600
C5—H5A	0.9700	C12—H12C	0.9600
C5—H5B	0.9700	C14—H14A	0.9600
C6—C16	1.522 (4)	C14—H14B	0.9600
C6—C1	1.527 (3)	C14—H14C	0.9600
C6—C7	1.549 (4)	C15—H15A	0.9600
C7—C16	1.510 (4)	C15—H15B	0.9600
C7—C8	1.510 (4)	C15—H15C	0.9600
C7—C13	1.516 (4)	C16—Cl1	1.766 (3)
C13—H13A	0.9600	C17—C22	1.513 (3)
C13—H13B	0.9600	C17—C18	1.521 (4)
C13—H13C	0.9600	C17—C27	1.574 (3)
C29—C23	1.526 (4)	C17—H17	0.9800
C29—H29A	0.9600	C18—O2	1.211 (3)
C29—H29B	0.9600	C18—C19	1.473 (4)
C29—H29C	0.9600	C19—C20	1.339 (4)
C30—C27	1.537 (4)	C19—C28	1.505 (4)
C30—H30A	0.9600	C20—C21	1.491 (4)
C30—H30B	0.9600	C20—H20	0.9300
C30—H30C	0.9600	C21—C22	1.509 (3)
C32—C23	1.504 (4)	C21—H21A	0.9700
C32—C22	1.513 (3)	C21—H21B	0.9700
C32—Cl4	1.756 (3)	C22—C23	1.539 (3)
C32—Cl3	1.766 (3)	C23—C24	1.517 (4)
Cl2—C16	1.764 (3)	C24—C25	1.523 (5)
C1—C2	1.515 (4)	C24—H24A	0.9700
C1—C11	1.576 (3)	C24—H24B	0.9700
C1—H1	0.9800	C25—C26	1.527 (5)
C2—O1	1.228 (3)	C25—H25A	0.9700
C2—C3	1.474 (4)	C25—H25B	0.9700
C3—C12	1.506 (4)	C26—C27	1.537 (5)
C8—C9	1.535 (4)	C26—H26A	0.9700
C8—H8A	0.9700	C26—H26B	0.9700
C8—H8B	0.9700	C27—C31	1.549 (5)
C9—C10	1.519 (4)	C28—H28A	0.9600
C9—H9A	0.9700	C28—H28B	0.9600
C9—H9B	0.9700	C28—H28C	0.9600
C10—C11	1.529 (4)	C31—H31A	0.9600
C10—H10A	0.9700	C31—H31B	0.9600
C10—H10B	0.9700	C31—H31C	0.9600
			
C3—C4—C5	125.3 (3)	C11—C14—H14A	109.5
C3—C4—H4	117.4	C11—C14—H14B	109.5
C5—C4—H4	117.4	H14A—C14—H14B	109.5
C4—C5—C6	111.8 (2)	C11—C14—H14C	109.5
C4—C5—H5A	109.3	H14A—C14—H14C	109.5
C6—C5—H5A	109.3	H14B—C14—H14C	109.5
C4—C5—H5B	109.3	C11—C15—H15A	109.5
C6—C5—H5B	109.3	C11—C15—H15B	109.5
H5A—C5—H5B	107.9	H15A—C15—H15B	109.5
C5—C6—C16	118.0 (2)	C11—C15—H15C	109.5
C5—C6—C1	113.9 (2)	H15A—C15—H15C	109.5
C16—C6—C1	117.1 (2)	H15B—C15—H15C	109.5
C5—C6—C7	121.5 (2)	C7—C16—C6	61.42 (18)
C16—C6—C7	58.90 (17)	C7—C16—Cl2	118.7 (2)
C1—C6—C7	116.6 (2)	C6—C16—Cl2	119.7 (2)
C16—C7—C8	118.4 (3)	C7—C16—Cl1	121.5 (2)
C16—C7—C13	119.3 (3)	C6—C16—Cl1	121.25 (19)
C8—C7—C13	113.7 (3)	Cl2—C16—Cl1	108.17 (15)
C16—C7—C6	59.67 (17)	C22—C17—C18	110.30 (19)
C8—C7—C6	116.4 (2)	C22—C17—C27	114.06 (19)
C13—C7—C6	119.2 (3)	C18—C17—C27	112.9 (2)
C7—C13—H13A	109.5	C22—C17—H17	106.3
C7—C13—H13B	109.5	C18—C17—H17	106.3
H13A—C13—H13B	109.5	C27—C17—H17	106.3
C7—C13—H13C	109.5	O2—C18—C19	120.1 (2)
H13A—C13—H13C	109.5	O2—C18—C17	120.4 (2)
H13B—C13—H13C	109.5	C19—C18—C17	119.5 (2)
C23—C29—H29A	109.5	C20—C19—C18	119.4 (2)
C23—C29—H29B	109.5	C20—C19—C28	123.1 (3)
H29A—C29—H29B	109.5	C18—C19—C28	117.5 (3)
C23—C29—H29C	109.5	C19—C20—C21	124.6 (2)
H29A—C29—H29C	109.5	C19—C20—H20	117.7
H29B—C29—H29C	109.5	C21—C20—H20	117.7
C27—C30—H30A	109.5	C20—C21—C22	112.3 (2)
C27—C30—H30B	109.5	C20—C21—H21A	109.1
H30A—C30—H30B	109.5	C22—C21—H21A	109.1
C27—C30—H30C	109.5	C20—C21—H21B	109.1
H30A—C30—H30C	109.5	C22—C21—H21B	109.1
H30B—C30—H30C	109.5	H21A—C21—H21B	107.9
C23—C32—C22	61.35 (17)	C21—C22—C17	113.7 (2)
C23—C32—Cl4	118.95 (19)	C21—C22—C32	117.3 (2)
C22—C32—Cl4	119.78 (18)	C17—C22—C32	118.1 (2)
C23—C32—Cl3	120.68 (18)	C21—C22—C23	120.8 (2)
C22—C32—Cl3	121.13 (18)	C17—C22—C23	117.5 (2)
Cl4—C32—Cl3	108.56 (14)	C32—C22—C23	59.02 (17)
C2—C1—C6	110.0 (2)	C32—C23—C24	119.1 (2)
C2—C1—C11	112.6 (2)	C32—C23—C29	119.7 (3)
C6—C1—C11	114.7 (2)	C24—C23—C29	112.3 (2)
C2—C1—H1	106.3	C32—C23—C22	59.62 (15)
C6—C1—H1	106.3	C24—C23—C22	116.7 (2)
C11—C1—H1	106.3	C29—C23—C22	120.0 (2)
O1—C2—C3	120.3 (2)	C23—C24—C25	112.4 (3)
O1—C2—C1	119.9 (2)	C23—C24—H24A	109.1
C3—C2—C1	119.7 (2)	C25—C24—H24A	109.1
C4—C3—C2	119.2 (3)	C23—C24—H24B	109.1
C4—C3—C12	123.0 (3)	C25—C24—H24B	109.1
C2—C3—C12	117.3 (2)	H24A—C24—H24B	107.9
C7—C8—C9	112.7 (3)	C24—C25—C26	115.7 (3)
C7—C8—H8A	109.1	C24—C25—H25A	108.3
C9—C8—H8A	109.1	C26—C25—H25A	108.3
C7—C8—H8B	109.1	C24—C25—H25B	108.3
C9—C8—H8B	109.1	C26—C25—H25B	108.3
H8A—C8—H8B	107.8	H25A—C25—H25B	107.4
C10—C9—C8	115.4 (2)	C25—C26—C27	119.7 (3)
C10—C9—H9A	108.4	C25—C26—H26A	107.4
C8—C9—H9A	108.4	C27—C26—H26A	107.4
C10—C9—H9B	108.4	C25—C26—H26B	107.4
C8—C9—H9B	108.4	C27—C26—H26B	107.4
H9A—C9—H9B	107.5	H26A—C26—H26B	106.9
C9—C10—C11	119.6 (3)	C30—C27—C26	110.6 (3)
C9—C10—H10A	107.4	C30—C27—C31	107.7 (3)
C11—C10—H10A	107.4	C26—C27—C31	107.3 (3)
C9—C10—H10B	107.4	C30—C27—C17	108.0 (2)
C11—C10—H10B	107.4	C26—C27—C17	111.6 (2)
H10A—C10—H10B	106.9	C31—C27—C17	111.7 (3)
C10—C11—C15	107.4 (2)	C19—C28—H28A	109.5
C10—C11—C14	110.0 (2)	C19—C28—H28B	109.5
C15—C11—C14	108.0 (2)	H28A—C28—H28B	109.5
C10—C11—C1	111.6 (2)	C19—C28—H28C	109.5
C15—C11—C1	112.4 (2)	H28A—C28—H28C	109.5
C14—C11—C1	107.4 (2)	H28B—C28—H28C	109.5
C3—C12—H12A	109.5	C27—C31—H31A	109.5
C3—C12—H12B	109.5	C27—C31—H31B	109.5
H12A—C12—H12B	109.5	H31A—C31—H31B	109.5
C3—C12—H12C	109.5	C27—C31—H31C	109.5
H12A—C12—H12C	109.5	H31A—C31—H31C	109.5
H12B—C12—H12C	109.5	H31B—C31—H31C	109.5
